# Decellularization of rat adipose tissue, diaphragm, and heart: a comparison of two decellularization methods

**DOI:** 10.3906/biy-1807-109

**Published:** 2018-12-10

**Authors:** Melis SESLİ, Esin AKBAY, Mehmet Ali ONUR

**Affiliations:** 1 Department of Biology, Faculty of Science, Hacettepe University , Ankara , Turkey

**Keywords:** Scaofld, decellularization, extracellular matrix, rat, adipose-tissue-derived mesenchymal stem cells

## Abstract

Decellularization is a process that involves the removal of cellular material from the tissues and organs while maintaining the structural, functional, and mechanical properties of extracellular matrix. The purpose of this study was to carry out decellularization of rat adipose tissue, diaphragm, and heart by using two different methods in order to compare their efficiency and investigate proliferation profiles of rat adipose-tissue-derived mesenchymal stem cells (AdMSCs) on these scaffolds. Tissues were treated with an optimized detergent-based decellularization (Method A) and a freeze-and-thaw-based decellularization (Method B). AdMSCs were then seeded on scaffolds having a density of 2 × 10^5^ cells/scaffold and AO/PI double-staining and MTT assays were performed in order to determine cell viability. In this study, which is the first research comparing two methods of decellularization of an adipose tissue, diaphragm, and heart scaffolds with AdMSCs, Method A provided efficient decellularization in these three tissues and it was shown that these porous scaffolds were cyto-compatible for the cells. Method B caused severe tissue damage in diaphragm and insufficient decellularization in heart whereas it also resulted in cyto-compatible adipose tissue scaffolds.

## 1. Introduction

Extracellular matrix (ECM) is a structure composed of many types of proteins and polysaccharides such as laminin, collagens, proteoglycans, and glycosaminoglycans (Hoshiba et al., 2010; Hoshiba et al., 2015) . The properties (e.g., composition, structure) of ECMs change according to each type of tissue and organ in terms of providing tissuespecific cellular functions (Sellaro et al., 2007; Hoshiba et al., 2010) . Ideally, in tissue engineering, a functional scaffold should be able to mimic the in vivo ECM not only to support cells but also to promote cell adhesion, migration, proliferation, and differentiation (Rigogliuso et al., 2012; Dunne et al., 2014) . Due to the difficulty of mimicking the complex in vivo ECM, naturally derived ECM is commonly used instead of synthetic scaffolds (Hoshiba et al., 2010; Gilpin and Yang, 2017) and this ECM needs to be decellularized before tissue engineering applications.

Depending on the characteristics of each tissue and organ, the process of decellularization is performed physically, chemically, enzymatically, or by using combinative methods. All of these methods have their advantages and limitations (Crapo et al., 2011) . The main objective of decellularization is to minimize the loss and damage of the key ECM components while maximizing the removal of cellular material (Crapo et al., 2011; Tapias and Ott, 2014) . After these decellularization processes, tissue- or organ-derived biological scaffold is used as a biomimetic scaffold for the engineering of tissues and organs such as adipose, diaphragm, heart, blood vessels, bone, skin, liver, lung (Chen et al., 2013; Cheng et al., 2014; Dunne et al., 2014; Price et al., 2014; Mazza et al., 2015; Sierad et al., 2015; Ferrando et al., 2016; Rana et al., 2017) .

Decellularized scaffolds that are seeded with stem cells are very promising for tissue engineering applications because stem cells are good cell sources for recellularization. AdMSCs are sources of mesenchymal stem cells that have a differentiation potential in mature adipocytes and other cell types (e.g., myocyte, chondrocyte, neurocyte, and osteoblasts) while they modulate the immune responses, vascularization, and the migration of host stem cells to the implantation site (Flynn, 2010; Dai et al., 2016) . There is no ethical issue related to these cells and they can be easily obtained. In this study, we aimed to obtain decellularized rat adipose tissue, diaphragm, and heart as naturally derived scaffolds in order to mimic the in vivo microenvironment. In addition, we also aimed to compare two different decellularization methods, Method A (Akbay and Onur, 2018) and Method B (Dunne et al., 2014) , for each desired organ/tissue and investigate the cell proliferation profile of AdMSCs on these decellularized scaffolds for future regenerative applications.

## 2. Materials and methods

### 2.1. Isolation and preparation of rat adipose tissue, diaphragm, and heart for decellularization

A total of five 250–300-g male Wistar albino rats were used after the approval (Permit no. 2012/52) of the Animal Care and Use Committee, Hacettepe University. The rats were euthanized via ether inhalation. Subcutaneous and gonadal adipose tissues, the entire diaphragm, and heart were isolated under sterile conditions and washed in sterile phosphate buffered saline 1X (PBS). The adipose tissues were sectioned into pieces of 1 × 1 cm, the diaphragms were sectioned in half (1 × 2 cm), and the hearts were longitudinally sectioned into four pieces (0.5 × 1 cm). The samples were then stored in PBS / 1% penicillin–streptomycin at +4 °C until decellularization processes.

### 2.2. Isolation, culture, and characterization of AdMSCs

Two male Wistar albino rats (250–300 g) were used for the isolation of AdMSCs after the approval of the Animal Care and Use Committee, Hacettepe University. After ether inhalation; subcutaneous and gonadal adipose tissues were collected under sterile conditions. Primary explant cell culture technique developed by our group was used for the isolation of AdMSCs (Niyaz et al., 2012) . Adipose tissues were placed into a transport medium (DMEM/F12 / 0.4% penicillin–streptomycin) and the tissues were cut into pieces (4–5 mm) in a petri dish. Tissue fragments were then transferred to a 6-well plate and incubated with primary medium (DMEM/F12 / 20% FBS / 0.2% penicillin–streptomycin) at 37 °C / 5% CO2. The culture medium was replaced every day and the flask surface was washed with sterile PBS to remove nonadherent cells until the stem cells reach confluence. Tissue fragments were then removed and these steps were repeated. When the cells reached the cozn fluency of 80%–90%, the culture medium was removed and the cells were harvested with 0.25% trypsin/EDTA. AdMSCs were passaged three times and cryopreserved for future applications. Cells from the second passage were used during experiments. MSCs were characterized by immunouflorescence staining of CD29, CD31, CD54, and CD90 molecules.

### 2.3. Decellularization

In this study, two different decellularization methods were used for adipose tissue, diaphragm, and heart, and these methods were compared. Table briefly represents each of these decellularization methods.

#### 2.3.1. Detergent-based decellularization (DT / METHOD A)

Decellularization steps were carried out at room temperature. The samples were treated with 0.5% sodium dodecyl sulfate (SDS) solution for two days; they were then exposed to 1% SDS solution for one day. After being washed with PBS, the samples were treated with 1% TritonX-100 solution for 1 h. The samples were washed with PBS at the end of each step. Decellularization samples were then rinsed in PBS and stored at +4 °C in PBS / 1% penicillin–streptomycin until lyophilization (Akbay and Onur, 2018) .

#### 2.3.2. Freeze-and-thaw-based decellularization (FT / METHOD B)

The samples were frozen at −80 °C for 30 min and thawed at room temperature for 15 min (three cycles) and washed in ultrapure water for two days. After freeze and thaw cycles, other decellularization steps were performed with 120 rpm agitation at room temperature. Then, the samples were treated with 0.5 M and 1 M NaCl for 4 h respectively and washed in ultrapure water overnight. The samples were then transferred into 0.25% trypsin/EDTA solution for 2 h and washed in distilled water for 1 h. After isopropanol treatment overnight, the samples were treated with 1% TritonX-100 for three days (daily solution change). Then, samples were washed in ultrapure water for two days, rinsed in PBS for one day, and stored at +4 °C in PBS / 1% penicillin–streptomycin until lyophilization (Dunne et al., 2014) .

### 2.4. Staining of the scaffolds

For histological analysis, nondecellularized control tissue samples and decellularized tissue scaffolds were fixed in 10% formalin for 8 h. After the fixation process, samples were prepared for Hematoxylin and Eosin (H&E), Masson’s Trichrome (MT), and DAPI stainings in order to observe the efficiency of decellularization before the cell seeding process.

### 2.5. Lyophilization

Decellularized samples were taken into Eppendorf tubes one day before the lyophilization process and were then frozen at –80 °C. After this step, the samples were dried at freeze dryer for one day and lyophilized samples were stored at −20 °C until the cell seeding process.

### 2.6. Sterilization and preparation of the scaffolds

Firstly, lyophilized scaffolds were transferred into 24well plates and exposed to ultraviolet (UV) irradiation for each side for 45 minutes. Secondly, the desired amount of parafilm were cut into pieces and soaked in 70% ethanol before coating the bottom of the 24-well plates. The wells were then coated with parafilm in order to prevent cell adhesion to the adhesive surface of the wells during the cell seeding process. These provided AdMSCs adhere only to the scaffold surfaces. After sterilizing the plates with 70% ethanol, plates were lastly exposed to 20 minutes of UV irradiation (Thevenot et al., 2008).

### 2.8. Cell seeding

**Figure 1:**
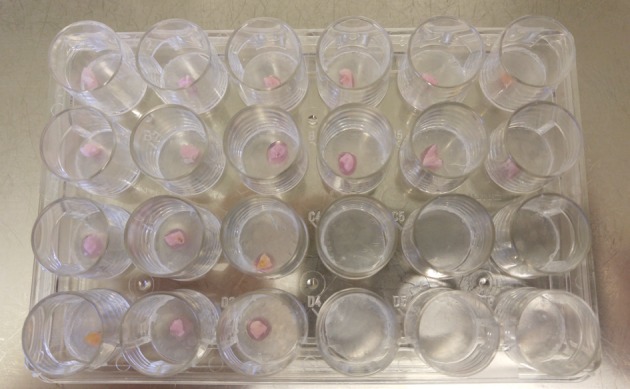
Decellularized tissue scaffolds placed in a parafilmcoated 24-well plate.

Taking into account the literature, 2 × 10^5^ cells for a 5 × 5-mm scaffold were determined (Wang et al., 2013a; Dunne et al., 2014) . Before the cell seeding process, AdMSCs from the second passage were trypsinized and cell suspension was prepared with DMEM/F12 / 10% FBS / 0.5% penicillin– streptomycin. Scaoflds were placed into parafilm-coated 24-well plates (one scaffold for each well; Figure 1). A cell suspension of 20 μL was seeded into each sample and allowed to incubate in a humidified incubator (37 °C / 5% CO2) for 2 h. Finally, in order to maintain 2 × 10^5^ cells/mL inoculation density for each scaffold, 200 µL medium was added ( Tığlı et al., 2009 ). After this step, the scaffolds were preserved in an incubator for 12 h. Then, a cell suspension of 300 µL was added on the scaffolds having a final volume of 500 µL for each well. These steps were also performed for decellularized control scaffolds with DMEM/F12 only. Finally, for MTT and AO/PI double-staining assays, the scaffolds were incubated at 37 °C / 5% CO 2 for 24, 48, and 72 h. During the cell seeding process, the cell suspension was incubated at 37 °C / 5% CO2 and pipetted before every usage.

### 2.9. MTT assay

MTT assay was used to evaluate cell viability quantitatively. At 24, 48, and 72 h, the culture medium was removed and the scaffolds were transferred to a new multiwell plate. A culture medium of 600 µL was added from the edge of the well, and then the MTT solution of 60 µL was added directly onto each scaffold. After an incubation period of 3 h at 37 °C / 5% CO , the medium with MTT solution was removed 2 from each well. Then, the isopropanol solution was added and each scaffold was crushed efficiently to dissolve the formazan crystals. The final solution in crystal violet color was transferred into a 96-well plate and optical density was measured at 570 nm by using a microplate reader. MTT assay was also applied to the scaffolds without cells as control and the obtained data were subtracted from the measured values.

Diaphragm and heart scaffolds decellularized with Method B were not taken into account in MTT assay due to the presence of cellular content in the heart and severe tissue damage in the diaphragm after decellularization.

### 2.10. AO/PI double-staining assay

AO and PI are nucleic staining dyes and double-staining of these dyes was performed to determine the cell viability on the scaffold material. At 24, 48, and 72 h, the culture medium was removed and the scaffolds were transferred to a new multiwell plate and they were washed with PBS (X1). 1:1 (v:v), AO/PI solution was prepared and 300 µL solution was added directly onto each scaffold with a waiting period of one min for cells to absorb the dye efficiently. Then, the dye was removed and scaffolds were washed with PBS (X3). The samples were then immediately observed under an inverted microscope (Olympus IX70 Inverted Microscope, Japan) with fluorescent attachment before the fluorescent color began to fade.

### 2.11. Statistical analysis

The results were presented as mean absorbance ± standard deviation (SD). The data were analyzed using Mann– Whitney U Test with IBM SPSS 23.0. The significance level was accepted as 0.05; therefore, P-values higher than 0.05 were considered nonsignificant.

## 3. Results

### 3.1. Adipose tissue, diaphragm, and heart decellularization and scaffold structure

**Figure 2:**
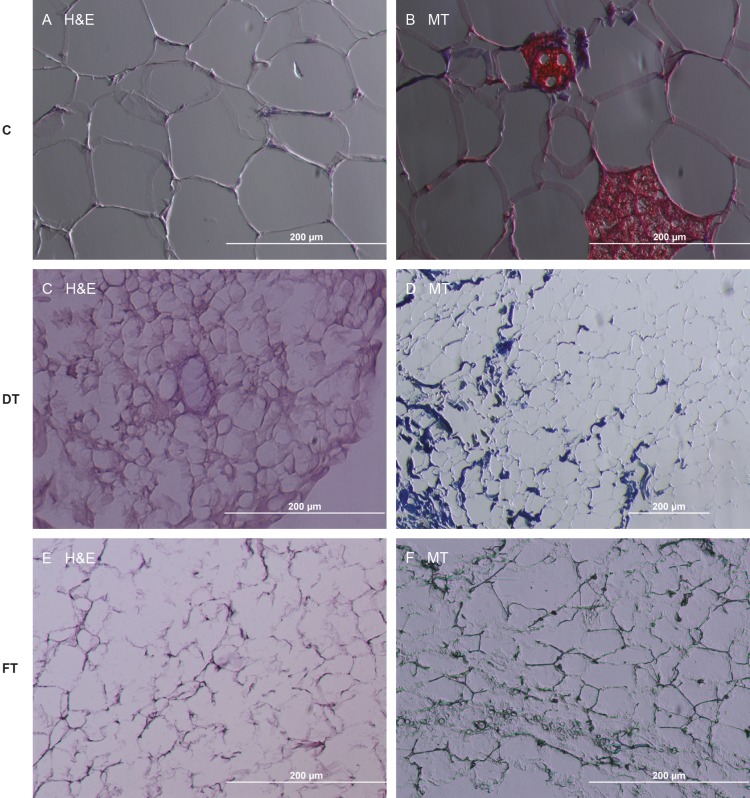
Histological image of nondecellularized and decellularized adipose tissue. Original magnification (100×). 10% formalin fixation was performed. (A, B) Nondecellularized control groups stained with H&E and MT; (C, D) Samples decellularized with Method A and stained with H&E and MT; (E, F) Samples decellularized with Method B and stained with H&E and MT. Tissue integrity was preserved better in Method A than Method B and no intact nuclei were observed in both methods. C: Control, DT: Detergent, FT: Freeze and thaw.

Histology of native adipose tissues demonstrated the eccentric and flattened nuclei, erythrocytes, and connective tissue around the blood vessels (Figures 2A and 2B). Whereas Method A produced white, dry, and fibrous material, Method B produced a white material having a wet, gel-like appearance. For any of the two methods, no nuclei were observed in the H&E staining (Figures 2C-F). Compared with Method B, tissue integrity was preserved more in Method A.

**Figure 3:**
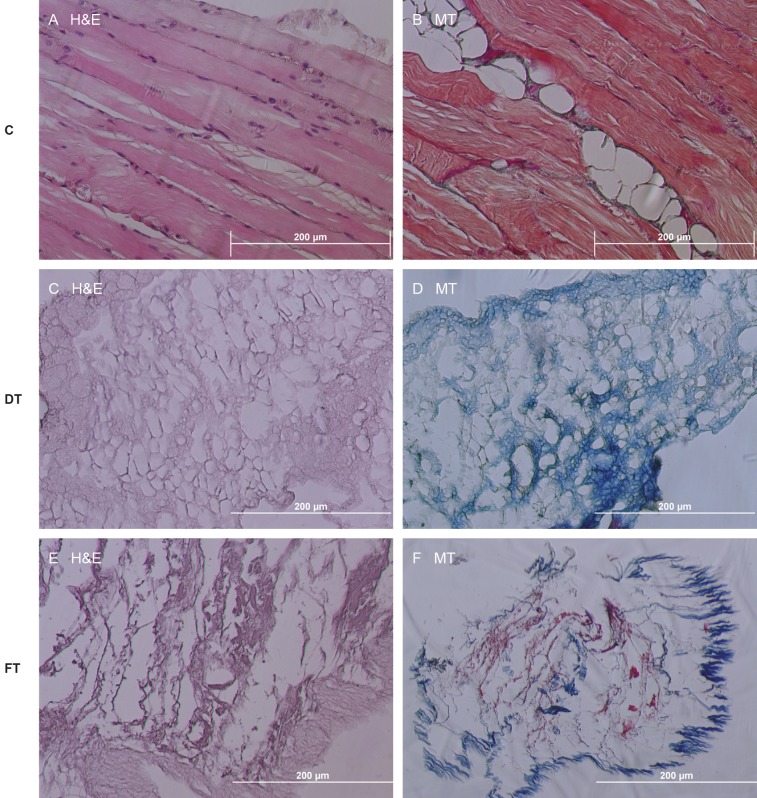
Histological image of nondecellularized and decellularized diaphragm. Original magnification (100×). 10% formalin fixation was performed. (A, B) Nondecellularized control groups stained with H&E and MT; (C, D) Samples decellularized with Method A and stained with H&E and MT. General tissue architecture was maintained in Method A. (E, F) Samples decellularized with Method B and stained with H&E and MT. Cellular content and severely impaired tissue integrity was observed. C: Control, DT: Detergent, FT: Freeze and thaw.

Native diaphragm histology demonstrated the intrafibrous connective tissue, large number of nuclei, and transverse striations (Figures 3A and 3B). During the decellularization with Method A and Method B, the diaphragms became translucent and dry, and white and fibrous materials were obtained after lyophilization. Whereas the decellularized diaphragms with Method A had no nuclei or muscle fibers (Figures 3C and 3D), cellular content was observed in the diaphragms decellularized with Method B (Figures 3E and 3F). While Method B caused severely impaired tissue integrity with degradation of collagen fibers, in Method A, substantial amounts of collagen were preserved and the general tissue architecture was retained as seen in MT staining.

**Figure 4:**
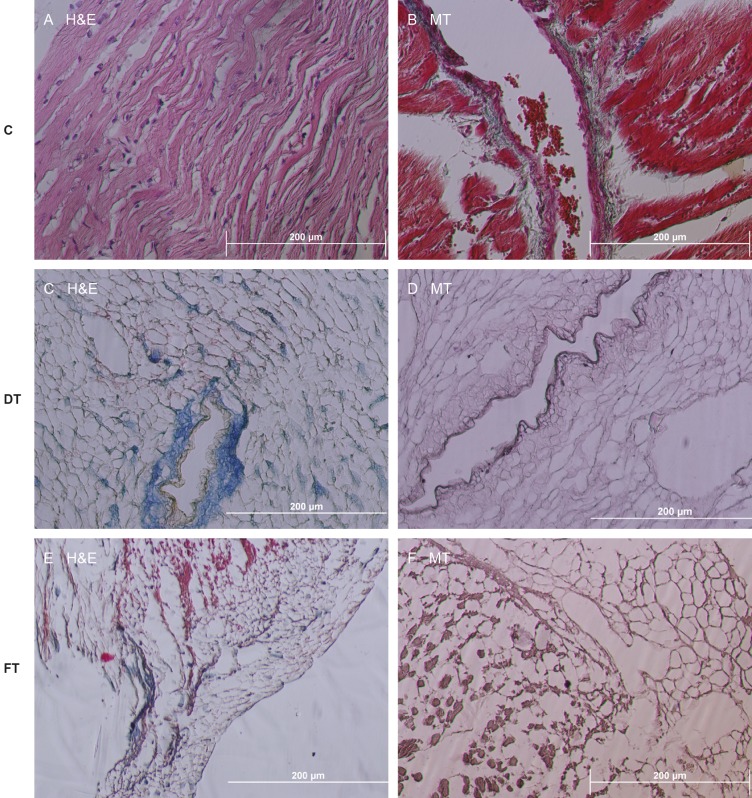
Histological image of nondecellularized and decellularized heart. Original magnification (100×). 10% formalin fixation was performed. (A, B) Nondecellularized control groups stained with H&E and MT; (C, D) Samples decellularized with Method A and stained with H&E and MT. Tissue integrity was preserved in Method A; (E, F) Samples decellularized with Method B and stained with H&E and MT. Insufficient decellularization was observed. C: Control, DT: Detergent, FT: Freeze and thaw.

Histology of native hearts demonstrated the striated appearance of the cardiac muscle, nuclei, blood vessels, and erythrocytes (Figures 4A and 4B). Heart tissues gradually became white in the decellularization with Method A, while the tissues were gray in Method B. Decellularized hearts with Method A had no intact nuclei or muscle fibers (Figures 4C and 4D) whereas cell nuclei and muscle fibers were observed in the hearts decellularized with Method B (Figures 4E and 4F). This situation indicates an insufficient decellularization in terms of Method B. When compared with Method B, collagen was maintained and general tissue architecture was preserved better in Method A.

### 3.2. Isolation and characterization of AdMSCs

**Figure 5:**
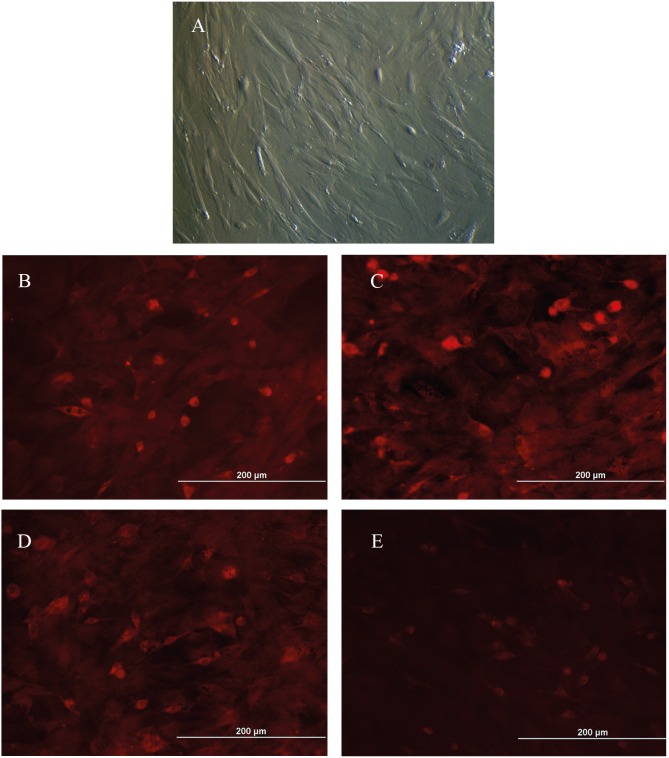
Inverted microscope image of rat AdMSCS, isolated by primary explant cell culture technique (A). Immunofluorescence staining showed that these cells were positive for surface markers CD29 (B), CD54 (C), and CD90 (D), and negative for CD31 (E).

The primary explant culture technique was performed for the cell isolation process. Within seven days, the cells showed a spindle-shaped fibroblastic cell morphology and reached confluency (Figure 5A). In order to identify AdMSCs, immunouflorescence staining was performed for the second passage. The staining showed that these cells were positive for surface markers CD29, CD54, and CD90, and negative for CD31 (Figures 5B–5E).

### 3.3. Cell proliferation in adipose tissue, diaphragm and heart scaffolds

**Figure 6:**
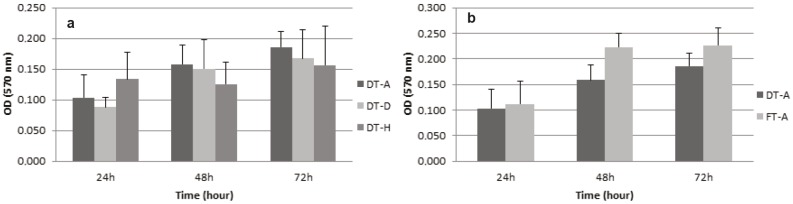
The proliferation profile of AdMSCs was measured by MTT assay. Data represents mean absorbance value. Error bars represent standard deviation. n = 3 for each decellularization method. (a) The proliferation profile of AdMSCs showed no statistically significant differences between adipose tissue, diaphragm, and heart scaffolds decellularized with Method A (P > 0.05). (b) The proliferation profile of AdMSCs showed no statistically significant differences between adipose tissue scaffolds decellularized with Method A and Method B (P > 0.05). DT: Detergent, FT: Freeze and thaw.

To compare the proliferation profile of AdMSCs, MTT assay was performed at 24, 48, and 72 h. Due to the presence of cellular content, severe tissue damage on diaphragm, and insufficient decellularization of the heart in Method B, these tissue scaffolds were not selected for the MTT assay. Comparison was performed only between Method A scaffolds as seen in Figure 6A. In adipose, diaphragm, and heart tissue scaffolds obtained by Method A, the proliferation of AdMSCs remained increasing at day three and no statistically significant differences were observed (P > 0.05). For the adipose tissue, the scaffolds obtained from Method A and B, the comparison was performed and it was observed that the proliferation of AdMSCs remained increasing for three days with no statistically significant differences between the decellularization methods (P > 0.05) (Figure 6B).

### 3.4. Cell viability of AdMSCs using AO/PI double-staining

**Figure 7:**
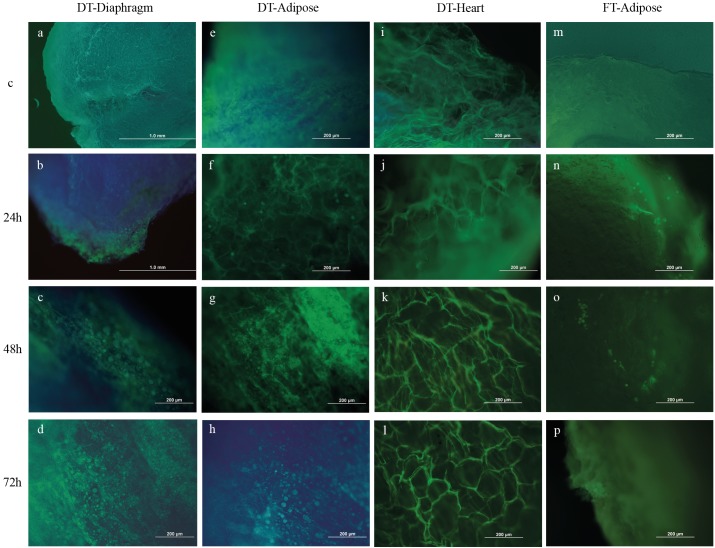
Dual fluorescence for viable and nonviable AdMSCs on adipose tissue (a–d), diaphragm (e–h), and heart (i–l) scaffolds decellularized with Method A, and adipose tissue decellularized with Method B (m–p) after 24, 48, 72 h. The presence of cell nuclei (light blue) was indicated by DAPI and cell viability (light green) was shown by AO/PI. When cultured on Method-A scaffolds, cells occupied the porous space within the nanofibrous structure and these scaffolds were cyto-compatible. Adipose tissue decellularized with Method B was also cyto-compatible for AdMSCs with a gel-like structure. AO/PI staining results matched with MTT assay results as seen in the images (original magnification 40× or 100×). C: Control, DT: Detergent, FT: Freeze and thaw.

Figure 7 shows the fluorescent images of AdMSCs on adipose tissue, diaphragm, and heart scaffolds, which were decellularized by the two different methods, after 24, 48, and 72 h. Most of the cells were viable and exhibited green light. No intact nuclei were observed on control tissue scaffolds. Porous structure of the scaffolds was observed in Method A. Wet, shiny structure of the adipose tissue scaffolds was observed in Method B.

## 4. Discussion

Successfully engineered tissues and organs should have several characteristics such as size and structural similarity to the native organ, similar biomechanical properties, nonimmunogenicity, and supporting healthy cell growth (Gubareva et al., 2016) . In the present study, we decellularized rat adipose tissue, diaphragm, and heart with two different decellularization methods: detergentbased method (Method A) (Akbay and Onur, 2018) and freeze-and-thaw-based method (Method B) (Dunne et al., 2014) . We also investigated the proliferation profiles of AdMSCs in these scaffolds. For the first time in the literature, these two methods were compared in terms of decellularization of adipose, diaphragm, and heart tissues and cell compatibility.

AdMSCs, a cellular component of adipose tissue, have a differentiation potential into mature adipocytes and other cell types (e.g., myocyte, chondrocytes, neurocyte and osteoblasts) while they modulate the immune responses, vascularization. and the migration of host stem cells to the implantation site (Flynn, 2010; Dai et al., 2016) . In this study, rat AdMSCs were isolated successfully by the primary explant cell culture techniques (Niyaz et al., 2012) and were characterized with immunouflorescence staining.

In tissue engineering, ECM scaffolds are ideal candidates because ECM supports the tissue and regulates cellular functions such as cell differentiation and proliferation. In this study, adipose, diaphragm, and heart tissues were decellularized by using two different techniques in search of a porous and cyto-compatible ECM scaffold.

As an abundant source of ECM, adipose tissue has been widely used in adipose tissue engineering (Flynn, 2010) . Dunne et al. (2014) used human adipose-tissuederived extracellular matrix (hDAM) for the investigation of breast cancer growth, and in another study, hDAM and human adipose-derived stem cells (hASCs) were combined to create a graft (Wang et al., 2013b; Dunne et al., 2014) . Though several methods were oefred and compared for adipose tissue decellularization (Brown et al., 2011) , in our study, two methods were performed. Eventually, Method A produced dry, white, fibrous, and porous scaffolds, whereas Method B produced white, wet, and gel-like scaffolds. Tissue structure was preserved more in Method A and impaired tissue integrity was observed in Method B. Proliferation of AdMSCs gradually increased both in Method A and Method B until day three and no statistically significant differences were found. AO/PI staining also supported these results.

Decellularized scaffolds are commonly used also in cardiac regenerative applications. For the heart tissue, there are many decellularization methods including immersion and perfusion decellularization and combinative methods (physical, chemical, and enzymatic) (Chang et al., 2002; Wei et al., 2006; Kajbafzadeh et al., 2017; Akbay and Onur, 2018) . In this study, two different decellularization methods were performed for the heart tissue. Method A resulted in a porous scaffold with preserved tissue integrity and no intact nuclei or muscle fibers were observed in this method. On the other side, in Method B, decellularization partially occurred. Therefore, MTT assay was only based on Method A scaffolds. Proliferation of AdMSCs sustained at day three by demonstrating a cyto-compatible scaffold structure. Well-protected tissue architecture can be observed in AO/PI staining with a much higher capacity of cell adhesion potential of AdMSCs, so the cells might have been in the depths of the tissue and therefore cannot be seen in flourescence imaging. When compared with adipose tissue and diaphragm scaffolds produced with Method A, no statistically significant differences were observed in the proliferation profile of AdMSCs.

Application of the decellularized patches is also preferred in skeletal muscle tissue engineering. Especially, surprisingly, the use of the diaphragm muscle has recently increased. Diaphragms can be decellularized by detergent– enzymatic methods and perfusion decellularization (Gubareva et al., 2016; Piccoli et al., 2016) . Compared with the previous studies, the difference of our study is testing the cell viability by seeding AdMSCs onto porous scaffolds which were obtained both by Method A and Method B. Whereas the tissue architecture and substantial amounts of collagen were maintained in Method A, Method B caused severe tissue damage and partial decellularization. Hence, MTT assay was performed only with scaffolds produced by Method A. Successful proliferation of AdMSCs remained increasing at day three as it can be fully observed in AO/ PI staining and there were no statistically significant differences in the proliferation profile of AdMSCs when compared with the adipose tissue and heart scaffolds produced with Method A.

Method A was an optimized decellularization technique for the heart tissue in our laboratory (Akbay and Onur, 2018) . In this study, it was tested whether it would give the same reliable results also for adipose tissue and diaphragm or not. As it was expected, for each of these tissues/organs, this method successfully produced fibrous and porous scaffolds with preserved ECM architecture that are cyto-compatible with AdMSCs as shown by AO/ PI double-staining and MTT analyses. Efficiency and reduced application time of this method may make it a preferred decellularization technique in tissue engineering approaches.

Method B severely impaired the tissue integrity in the diaphragm and caused relatively less damage in the adipose and heart tissues. Although the procedure was drastic and the application was time-consuming in Method B, it was not efficient enough to remove the cells and the cellular content in the diaphragm and heart. In another tissue engineering study, this method was tested for human adipose tissue (Palpant and Metzger, 2010) and successful results were obtained. Considering these reliable results, this method was chosen in our study to be tested, and effective decellularization and preserved tissue architecture were also expected especially for rat diaphragm and heart. However, due to the specific ECM properties and cell behaviors for each tissue/organ, by using Method B, we obtained different results leading us to consider optimizing this method in terms of reducing the time and increasing the applicability and effectiveness as an alternative decellularization method for these organs. Again, contrary to the expectations, this method produced wet and gel-like scaffolds with a successful decellularization for rat adipose tissue. As seen with an increased proliferation rate on MTT assay, its compatibility for AdMSCs may lay the groundwork for further research on soft tissue repair.

In conclusion, it was observed that Method A helps maintain native ECM properties such as porous structure in adipose tissue, diaphragm, and heart and these scaffolds can mimic the native microenvironment of the desired tissue/organ. Method A is more efficient in removing the cellular content from the desired tissue/organ within the shortest processing time. Reduced processing time can be cost-effective and useful for applicability of the method. Scaoflds produced with Method A and adipose tissue scaffolds produced with Method B are cyto-compatible and can be successfully recellularized with AdMSCs. Method B produces partially decellularized scaffolds for diaphragm and heart and damages the tissue integrity. Wet and gel-like appearance of adipose tissue decellularized with Method B may represent a possible hydrogel-like structure as a volume-filling construct for soft tissue repair or an injectable tissue filler to be investigated in future studies. Time frames for MTT assay could be extended to 14–21 days in order to obtain more reliable results for proliferation profile of AdMSCs on these scaffolds. These decellularized materials may be enlarged depending upon the animal model, tissue/organ, and the need of repair. These results may lead us to replicate this study in the future with the organs or tissues of a larger animal model such as porcine or human as a reliable support on the way of clinical setting.
